# A local outpatient practice-level prediction model for short-term psychiatric emergency presentation

**DOI:** 10.1093/jamiaopen/ooag215

**Published:** 2026-11-02

**Authors:** John L Havlik, Ben Tyrrell, Jeanette Polaschek, Nathan Bell, Eric R Arzubi

**Affiliations:** Department of Psychiatry and Behavioral Sciences, Stanford University School of Medicine, Palo Alto, CA 94304, United States; Big Sky Care Connect, Missoula, MT 59802, United States; Big Sky Care Connect, Missoula, MT 59802, United States; Frontier Psychiatry, Billings, MT 59102, United States; Big Sky Care Connect, Missoula, MT 59802, United States; Frontier Psychiatry, Billings, MT 59102, United States; Yale Child Study Center, Yale University School of Medicine, New Haven, CT 06519, United States

**Keywords:** health information exchange, machine learning, mental health services, emergency services, psychiatric, risk assessment

## Abstract

**Objectives:**

To determine whether statewide health information exchange (HIE) data can support practice-level prediction of short-term psychiatric emergency presentation, and to compare a locally trained model with general-purpose clinical risk scores.

**Materials and Methods:**

Retrospective cohort of patients at 1 Montana psychiatry practice with records in the Big Sky Care Connect statewide HIE. Structured HIE data were linked to zip code-level Social Vulnerability Index and Area Deprivation Index measures. Each observation was a patient snapshot. Logistic regression, random forest, and LightGBM models were tuned with 5 × 5 nested cross-validation and evaluated on a temporally held-out test set. The primary outcome was psychiatric or substance-related emergency department (ED) presentation within 30 days. Discrimination was compared with a parsimonious clinical baseline and the LACE and Elixhauser indices.

**Results:**

The test set contained 16 469 snapshots, of which 343 (2.1%) preceded a qualifying ED presentation (102 visits among 68 patients). A random forest model achieved area under the receiver-operator characteristic curve 0.85 (95% CI, 0.75-0.91). At a threshold targeting one-third recall, precision was 0.29 (0.12-0.44) and number needed to evaluate 3.4 (2.3-8.1). Discrimination exceeded the clinical baseline (Δ area under the curve 0.10), LACE (0.19), and Elixhauser (0.27); all *P* values less than  .001.

**Discussion:**

Structured HIE data, without clinical notes, supported a practice-specific model that outperformed general-purpose scores such as LACE and Elixhauser. Wide CIs reflect a small event count and constrain point-estimate claims.

**Conclusion:**

health information exchange infrastructure may allow participating practices to build locally calibrated psychiatric risk models. Prospective study of clinical utility is needed.

## Introduction

Predicting psychiatric emergencies is a large and unsolved problem. As of now, care structures are largely focused on acute, expensive, and reactive care.^1^ Psychiatric emergency department (ED) visits are a trying and often very expensive experience for patients and payors. It is possible that many of these ED visits reflect issues which could have been treated in an outpatient setting, but have spiraled to the point that serious harm may occur and emergency psychiatric evaluation and potential inpatient stabilization is warranted. Psychiatric patients often have a difficult time advocating for appropriate care in the outpatient setting because navigating the fragmented, “ghost provider” rich^2^ and narrow^3^^,^^4^ mental health-care network landscape is challenging. Predicting psychiatric emergency needs to enable proactive response would thus be a welcome solution to a pressing societal problem.

There are limited attempts in the literature to solve the problem of predicting psychiatric emergencies. Some recent adjacent literature has attempted to predict mental health symptom risk after general inpatient hospitalization.^5^ While there are more general estimates of comorbidity and readmissions risks such as the Elixhauser score^6^ or LACE index^7^ that are commonly used to risk stratify patients, psychiatric-specific risk stratification remains unaddressed. Risk stratifying to figure out when to proactively intervene before psychiatric emergencies occur would mark a major shift in the field’s abilities to provide timely and value-based care to patients who need it most, before harm occurs.

Recent necessary advances on both the technical and health-care infrastructure fronts may have enabled the creation of such a tool. In machine learning, advances include interpretable and highly effective supervised learning techniques such as logistic regression, random forest modeling, and gradient boosting. Such machine learning techniques may enable meaningful insights into patient risk even when trained using only patients receiving care from a single specialty practice. Across national health-care infrastructure, key industry stakeholders from across the provider, payor, and government sectors have begun to facilitate interoperability of electronic health-care records data across a variety of health-care settings through the creation of state and regionally based health information exchanges (HIEs), organizations which aggregate and share patients’ health-care data across health-care systems and providers. Thus, a given patient’s data are made more complete, normalized, and synchronized across participating health-care systems and providers in a given state. In Montana, Big Sky Care Connect (BSCC) was founded in 2018 as a 501(c)(3) nonprofit to respond to the need for a statewide coordinated health information exchange. Big Sky Care Connect is the state designated HIE in Montana and shares health-care data across hospitals, clinics, federally qualified health centers (FQHCs), pharmacies, and other health-care settings throughout the state.^8^ Big Sky Care Connect is an opt-out provider of health information with 3120 unique providers (including physicians, nurses, and other clinical staff) and 1 702 991 unique patients served as of May 2026.

### Objective

The present paper describes the development and evaluation of a supervised machine learning model to predict 30-day psychiatric ED visits, defined as an emergency visit with any primary psychiatric or substance use International Classification of Diseases, Tenth Revision (ICD-10) code. This model was trained on HIE data from patients receiving care from a general psychiatry practice in Montana (Frontier Psychiatry). Because psychiatric emergency risk is shaped by local care networks and service availability, we designed this study as a proof of concept for local prediction modeling rather than as development of a universally transportable model. Accordingly, our objective was to assess whether routinely available local HIE data could support short-term risk stratification within this specific care setting. We emulated a prospective trial of our model by training on all practice patients entering the HIE before a certain date, and testing on all patients entering the HIE after this date. We hypothesized that our model would outperform general-purpose readmission and comorbidity indices on this task. We further sought to determine whether any predictive advantage from higher-dimensional machine learning models would justify their added complexity relative to a parsimonious clinical model.

## Methods

### Institutional approval and participant consent

This study received Institutional Review Board approval from the WIRB-Copernicus Group Inc. institutional review board. Informed consent was not required as data were retrospective and deidentified according to standard privacy guidelines; no patients or the public were involved in study design. This study followed the TRIPOD+AI reporting guideline for the development and validation of prediction models in health care.^9^

### Data sources and study population

To develop predictive models for 30-day emergent psychiatric presentations, we constructed a retrospective cohort using multisource clinical data of all patients seen by Frontier Psychiatry with records in the BSCC HIE database. This dataset contains data points such as patient encounters, labs, vitals, and other data. A full data dictionary for the 336 variables derived from the BSCC data used in this study can be found in Table S1. Cognizant of place-based disparities in psychiatric access, we supplemented this HIE data with 2 established geospatial datasets, the Social Vulnerability Index (SVI)^10^ and Area Deprivation Index (ADI),^11^ linking patients to SVI and ADI values based on zip code values from the HIE. Each observation consisted of a patient “snapshot,” defined as all structured data available up to a given index date. Final sample size, number of snapshots, and event counts are reported in the “Results” section.

### Anchor date, index date, and snapshot definitions

We defined each patient’s anchor date in the HIE as the patient’s earliest recorded health-care system interaction across all available structured data sources, including encounters, laboratory results, medication prescriptions, vital signs, and diagnoses. Patients were then assigned to temporally distinct cohorts using a cutoff date corresponding to the 90th percentile of anchor dates: a training cohort comprising patients with any anchor dates before June 5, 2023, and a test cohort comprising patients with anchor dates on or after June 5, 2023. This patient-level temporal split, based on HIE data available through October 30, 2025, was chosen to approximate prospective deployment and reduce information leakage^12^^,^^13^; accordingly, no patient contributed snapshots to both training and test sets. This temporal split was chosen over a random split protocol due to TRIPOD guidance which notes that a nonrandom temporal split provides a higher level of evidence for validation.^14^ Outcome prevalence in the test cohort was left unchanged to provide realistic estimates of model performance.

We transformed each patient’s longitudinal record into multiple prediction snapshots, generating 1 snapshot for each encounter day that met minimum observation criteria. Each snapshot was defined as a date a patient had an encounter with a health-care provider, with all predictors derived exclusively from data recorded before that index date. All encounters were eligible to serve as index events. Details on snapshot exclusions can be found in Material S1.

### Primary outcome definition

The primary outcome was an ED presentation for a psychiatric or substance use-related condition within the subsequent 30 days. Emergency department presentations were identified using encounter‑type fields in the HIE “Encounter” datafile indicating an encounter type of “Emergency.” We defined an encounter for psychiatric or substance use reasons as an encounter with a primary ICD-10 diagnosis codes outlined in Material S1.

### Secondary benchmarking definitions

Secondary benchmarks included the LACE index (used to predict 30-day hospital readmissions) and Elixhauser comorbidity index (a metric of composite disease burden used to predict and 30-day hospital readmissions), which were selected as established general-purpose measures of hospital readmission risk and comorbidity burden. Because general-purpose indices such as LACE and Elixhauser are not designed for psychiatric emergency prediction, we also specified a parsimonious “clinical baseline” model using a small set of face-valid predictors available in routine practice based on study team consensus. Predictors were age, sex, race, ethnicity, number of mental health diagnoses in the prior 90 days, any psychiatric ED visits in the prior 90 days, and total health-care system encounters in the prior 90 days. This logistic regression model was tuned, isotonic scaled, and evaluated within the same nested cross-validation and temporally held-out testing framework as the primary model.

### Feature engineering

We derived candidate features from structured data available before each snapshot index date using a domain-based feature engineering pipeline designed to prevent temporal leakage. Features were grouped into utilization, diagnostic, pharmacy, physiology/laboratory, demographic, and social determinant domains. Missing values were retained during feature construction and addressed subsequently within model-specific preprocessing. We did not select a subset of features for use in the final model, instead retaining all 336 features engineered. See Material S1.

### Model comparator specification

Before evaluation in the temporally held-out test set, we specified the primary outcome, candidate comparator models, and the model operating point. First, we developed a portfolio of machine learning models, including Logistic Regression, Random Forest, and LightGBM, to capture linear, bagging, and boosting signal structures, respectively. To tune hyperparameters and conduct preliminary performance testing for all models, we utilized a 5 × 5 nested cross-validation framework^15^^,^^16^ on the training set with limited hyperparameter grid search performed on the inner folds for all models. See Material S1.

### Model testing

Our operating goal was to catch the top third of emergency psychiatric presentations while maximizing precision. This operating point target was informed by Frontier’s capacity for proactive outreach and consistent with recommendations that prediction thresholds be aligned with intended clinical use and intervention burden.^17^ We determined our testing decision threshold using the hyperparameter tuning described above, plus an additional temporal adjustment for trailing 180-day event prevalence. Once our final model was selected, we tested our hyperparameter-tuned classifier on our temporally separated testing data; details of this testing are outlined in Material S1.

To measure clinical utility, we performed decision curve analysis to quantify net benefit across a range of decision thresholds, with specifications outlined in Material S1. To provide an interpretable window into the risk of algorithmic bias, we assessed model performance (operationalized by area under the precision-recall curve [PR AUC]) stratified by sex, race, ethnicity, and age band, with a prespecified subgroup sensitivity analysis accounting for differences in base rate prevalences. We additionally interrogated top model features using SHapley Additive exPlanations (SHAP) values to generate global beeswarm and local waterfall plots.

We conducted a limited set of statistical analyses in this manuscript, using DeLong’s test for correlated ROC curves^18^ to compare model performance between our final model, our parsimonious clinical baseline model, and LACE and Elixhauser scores (using their risk scores mapped to decisions across the same threshold grid), across all snapshots of patients with available risk score data. Significance was determined using a *P* < .05, Bonferroni-Holm adjusted at the comparison group level.^19^ All CIs reported in this study were generated via 1000 bootstrap iterations with replacement, clustered at the patient level, to account for correlation among snapshots from the same patient (repeated measures). Intervals reported thus reflect the number of patients rather than the number of snapshots. Differences between models were computed on the same resampled patients. Python version 3.14.3 was used for all analyses and for figure creation.

## Results

### Study population

The analytic unit for this study was the patient snapshot. Our final study population consisted of 11 463 patients in our training set and 1739 patients in our test set, comprising 143 203 training snapshots and 16 469 testing snapshots (Table 1, Figure S1). Our overall sample was 62.8% female, had a median (interquartile range) age of 36.6 (27.8-49.0), and had a median of 0 (0-0) emergency psychiatric presentations in the last year. In the test set, 343 snapshots (2.1%) were followed by a qualifying psychiatric ED presentation within 30 days, corresponding to 102 ED visits among 68 unique patients. Because patients could contribute multiple snapshots and multiple snapshots could precede the same event, snapshot-level event counts exceed unique event counts.

**Table 1.** Patient demographics and event information.

Variable	Training set (%)	Prospective test set (%)
**Total patient, *n***	11 463	1739
**Total psychiatric ED visit, *n***	1937	102
**Median (IQR) psychiatric ED visits**	0 (0-0)	0 (0-0)
**Snapshot date range**	January 1, 2022-June 4, 2023	December 4, 2023-June 30, 2025
**Median (IQR) snapshots per patient**	7 (3-16)	5 (2-11)
**Patient sex**		
** Female**	7313 (63.8)	979 (56.3)
** Male**	4133 (36.1)	760 (43.7)
** Missing**	17 (0.1)	0 (0.0)
**Age at index (years), median (IQR)**	36.5 (27.7-48.9)	37.5 (29.0-50.0)
**Race**		
** White**	8509 (74.2)	1173 (67.5)
** Asian**	40 (0.3)	20 (1.2)
** Other**	916 (8.0)	223 (12.8)
** Declined**	86 (0.8)	42 (2.4)
** Missing**	1922 (16.8)	281 (16.2)
**Ethnicity**		
** Hispanic or Latino**	420 (3.7)	50 (2.9)
** Not Hispanic or Latino**	9787 (85.4)	1304 (75.0)
** Declined**	37 (0.3)	14 (0.8)
** Missing**	1219 (10.6)	371 (21.3)
**Diagnosis (last 365 days)**		
** Anxiety disorders**	2235 (19.5)	793 (45.6)
** Depressive disorders**	1993 (17.4)	597 (34.3)
** Psychotic disorders**	331 (2.9)	76 (4.4)
** Bipolar disorders**	626 (5.5)	162 (9.3)
** Substance use disorders**	1887 (16.5)	316 (18.2)
** SI/Self-harm**	439 (3.8)	40 (2.3)
** Other mental health disorders**	2444 (21.3)	816 (46.9)

All characteristics reported at the patient level, not the snapshot level, at the patient’s first prediction date. Psychiatric disorders included ICD-10 F-codes F00-F99. Disorders included in anxiety disorders included F-codes F40-F41. Disorders included in depressive disorders included F-codes F32-F33. Disorders included in psychotic disorders included F-codes F20-F29. Disorders included in bipolar disorders included F-codes F30-F31. Disorders included in substance use disorders included F-codes F10-F19. Codes included in SI/Self-harm included ICD-10 codes R45.851 (suicidal ideation) and X71-X83 (intentional self-harm). Other mental health disorders included all other F-code diagnoses. Because ED visits for psychiatric reasons are rare events, the median number of ED psychiatric visits in our patient population was zero.

Abbreviations: ED, emergency department; IQR, interquartile range.

### Model selection

Our best-performing model on preliminary nested cross-validation was a random forest model, see Table S2 for preliminary cross-validation testing performance of all 3 models at optimized hyperparameters. This model had a mean ± SD precision at 1/3 recall of 0.22 ± 0.05 on our 5-fold outer-fold cross-validation testing, with a mean ± SD area under the receiver-operator characteristic curve (ROC AUC) of 0.78 + 0.02 and PR AUC of 0.22 ± 0.07. The modal hyperparameter settings for this model across outer folds were a class weight of null, a max tree depth of 12, a maximum feature setting of the square root of all features, a minimum samples per leaf setting of 4, and an estimator number setting of 500.

### Performance on unseen test data

In a prospective trial on all patients with anchor dates after the cutoff date, we applied the mean decision threshold which gave us 1/3 recall during the outer folds of our cross-validation testing. At this threshold, the model had a precision ± bootstrapped SD (95% CI) of 0.29 (0.12-0.44) and identified 49.3% (21.1%-69.8%) of emergency psychiatric visits. This model would need to flag 3.4 (2.3-8.1) patients to identify 1 true positive emergency psychiatric visit within the next 30 days; full model metrics can be seen in Table S3. Our prospectively trialed model had a ROC AUC of 0.85 (0.75-0.91) and PR AUC of 0.29 (0.08-0.48) on unseen test data. At this threshold, our model was 97.5% (96.6%-98.3%) specific and generated 3.5 (2.1-5.3) alarms per 100 patients (Figure 1). A sensitivity analysis using 1 random snapshot per test patient produced qualitatively similar performance metrics, though with wider CIs reflecting low *n* without snapshotting, ROC AUC 0.92 (0.70-0.94), Table S4.

**Figure 1.**
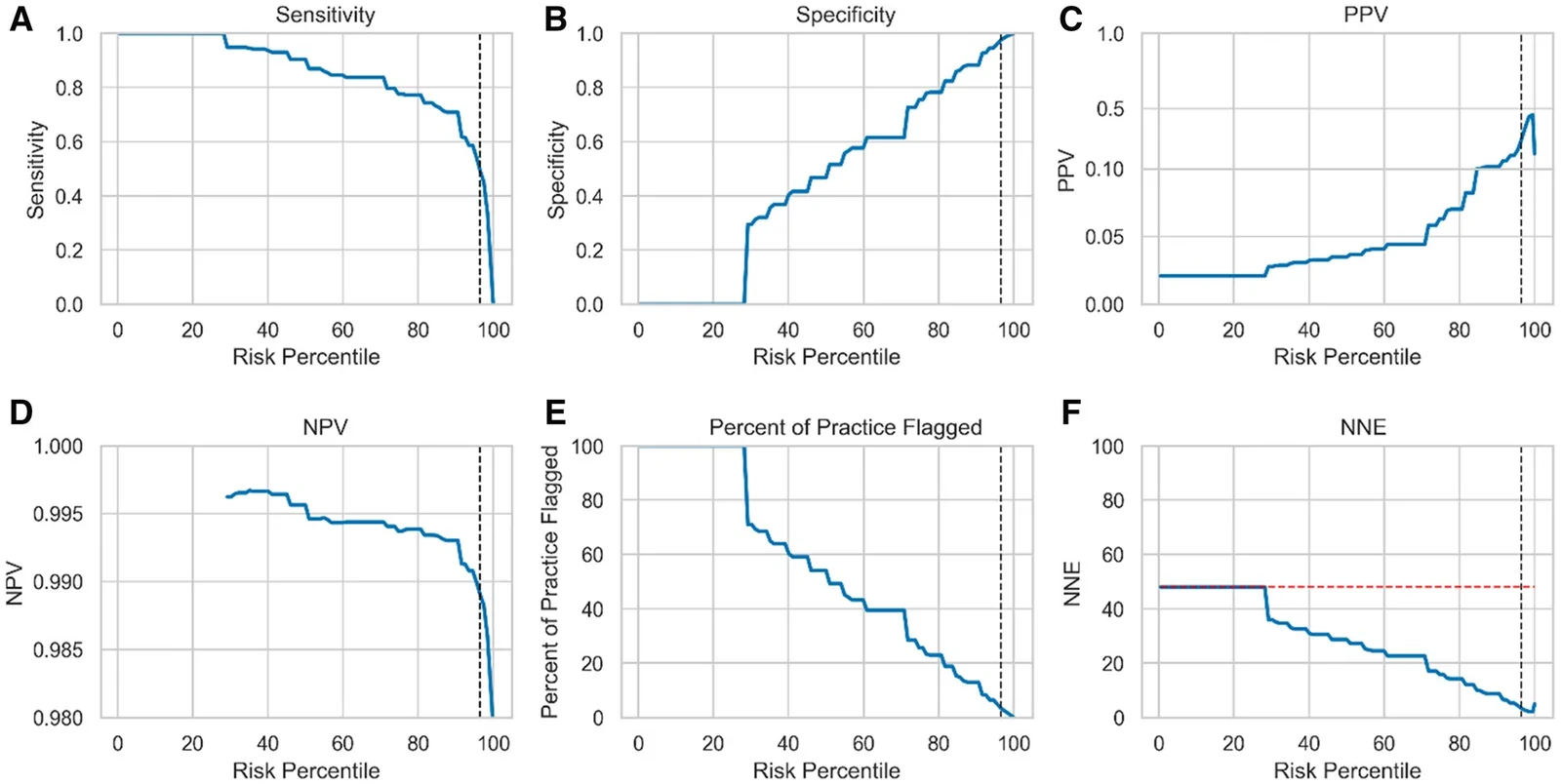
Model metrics across calculated risk percentiles. (A) Sensitivity on the held‑out test set as a function of risk percentile (higher percentiles correspond to more stringent thresholds). Vertical lines throughout this and subsequent figures mark sensitivity targets at prespecified decision threshold (dashed) targeting 1/3 recall. (B) Specificity vs risk percentile. (C) PPV vs risk percentile shown on split *y*‑axes to reveal detail at low values (lower pane: 0-0.1; upper pane: 0.1-1.0). (D) NPV vs risk percentile. (E) Percent of practice flagged (alarms per 100 patients) vs risk percentile. (F) NNE vs risk percentile, with a red dashed no‑skill reference equal to 1/prevalence; lower values indicate fewer false positives per true positive. Abbreviations: NNE, number needed to evaluate; NPV, negative predictive value; PPV, positive predictive value.

### Calibration analysis

Our calibration testing indicated our model’s ability to correctly gauge clinical risk was reasonable though imperfect on the test set (Brier = 0.017; slope = 1.36; intercept = 1.08). In the low‑probability region (0-0.03), observed event rates tracked predicted probabilities, but global metrics indicated some overconfidence at higher probabilities (Figure 2A).

**Figure 2.**
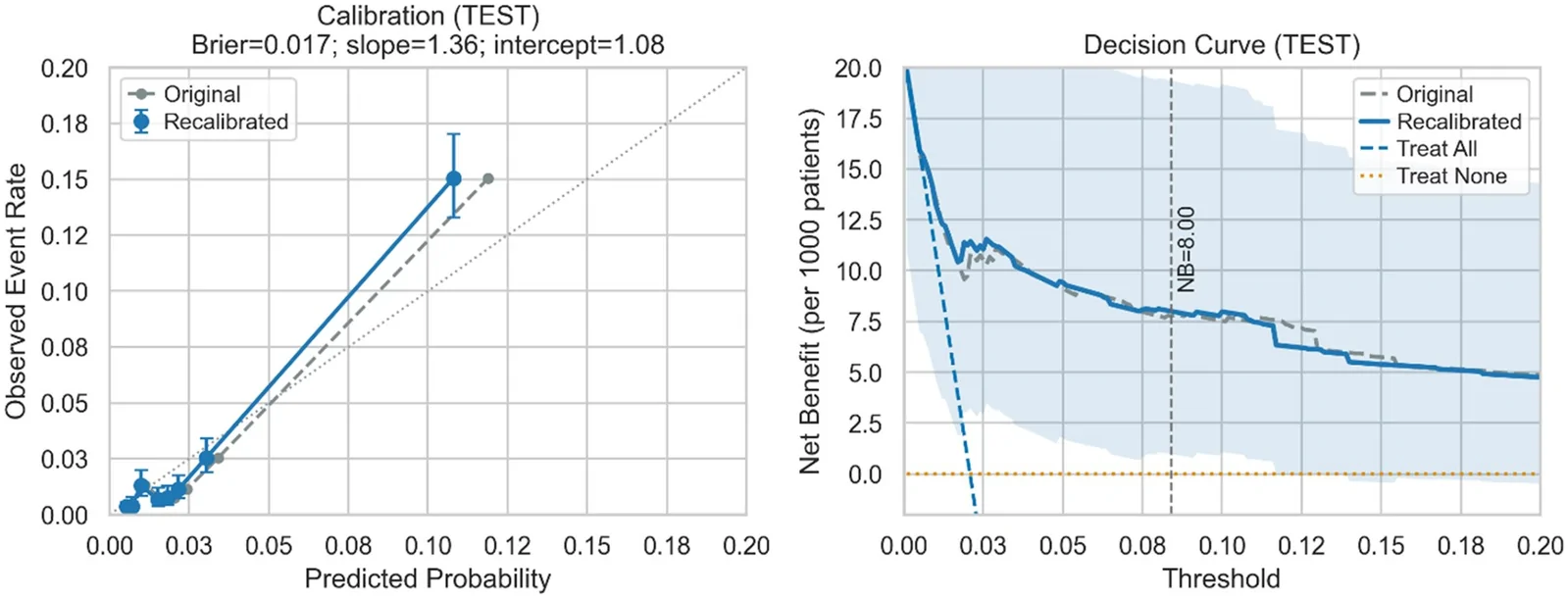
Model decision and calibration curves. (A, left) Decision curve analysis on the test set shows net benefit per 1000 patients as a function of threshold in the low-risk range. Vertical line indicates operating point at prespecified decision threshold; this decision threshold implied a cost: benefit ratio of 0.09, meaning the cost of a false positive alarm was valued at approximately 11 times less than the benefit of a true positive alarm. Net benefit is calculated as true positives per patient minus preference‑weighted false positives per patient, scaled to 1000 patients; higher values indicate greater clinical utility. Light blue shading reflects the bootstrapped 95% CI on the recalibrated test curve; the original (train‑prior) curve is shown as a dashed reference. (B, right) Calibration panel which shows observed event rates vs predicted probabilities with 95% Wilson CIs; the recalibrated model tracks the 45° line in the low‑probability region. Abbreviation: NB, net benefit. .

### Clinical utility: decision curve analysis

On decision curve analysis, our prespecified decision threshold implied a cost: benefit ratio of 0.09, meaning the cost of a false positive alarm was valued at approximately 11 times less than the benefit of a true positive alarm. At this operating point, the model’s net benefit was 8.0 per 1000 patients (95% CI, 1.0-19.4) relative to a baseline of no detection system. Decision curves were similar whether or not we a priori recalibrated our decision threshold for trailing 180-day event prevalence (Figure 2B).

### Subgroup analysis and model interpretability

Overall model performance was within 95% CIs for PR AUC for all subgroups except male sex and those ages 18-24 (the youngest age group in our sample) and 65 + (the oldest age group in our sample), Figure 3. In these groups, the model’s discriminatory ability was worse than average across the entire cohort. These differences in discriminatory ability were robust to subgroup analyses using prevalence‑adjusted average precision in all cases (Figure S2).

**Figure 3.**
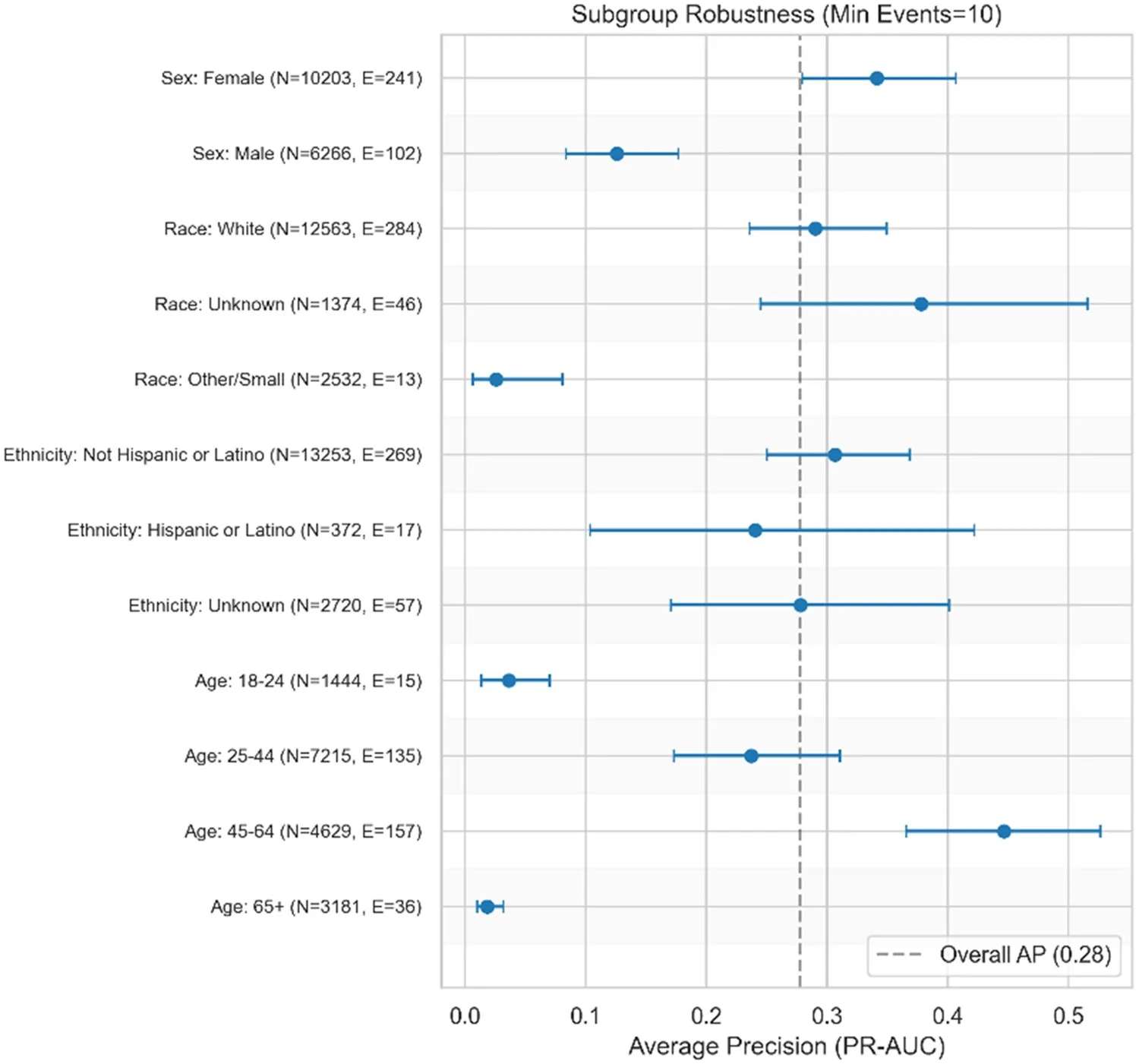
Model subgroup robustness analysis. Subgroup analysis conducted on age, sex, and race at the snapshot level; event denotes a snapshot with a 30-day emergency psychiatric visit. Points show subgroup performance with bootstrapped 95% CIs. Dashed line denotes overall standardized average precision (sAP). Higher values indicate better performance; sAP = 0 corresponds to random ranking. Prevalence-adjusted estimates of subgroup performance can be seen in Figure S2 .

Alongside this modeling, we conducted SHAP analysis to determine feature importance. Our models revealed top contributing features outlined in Figure 4. Notably, measures of utilization made up 14 of the 20 most important features in our model.

**Figure 4.**
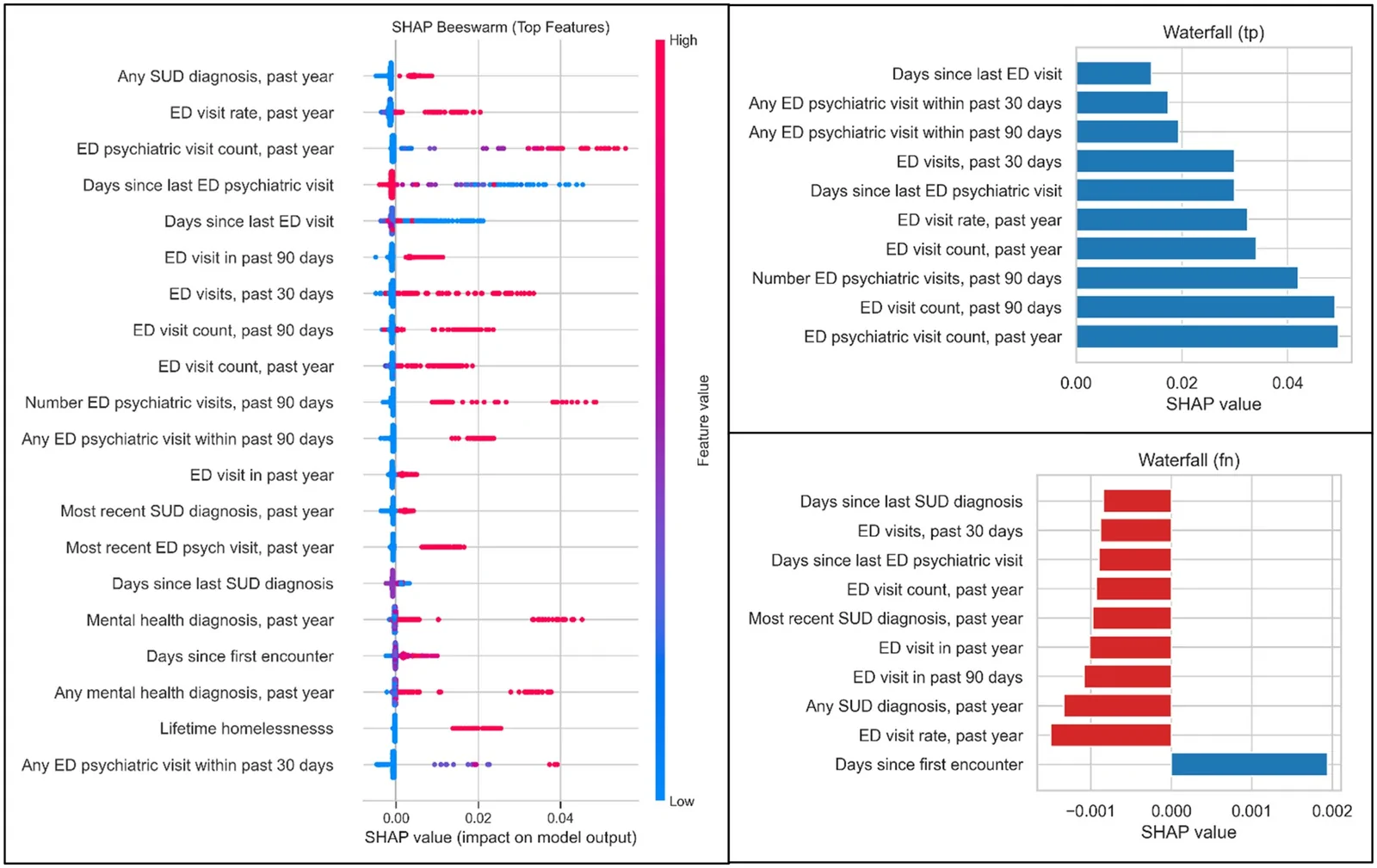
Model SHAP analysis. (A, left) SHAP beeswarm on the held‑out test set shows the distribution of feature contributions for the top predictors; features are ordered by mean absolute SHAP, dot color encodes feature value, and points to the right indicate contributions toward higher predicted risk. (B, top right) Waterfall plot for a representative high‑risk TP and for (C, bottom right) a missed FN highlight the top contributing features and their direction of effect for each case; bars to the right increase predicted risk and bars to the left decrease predicted risk, illustrating features behind individual predictions. Abbreviations: ED, emergency department; FN, false negative; SHAP, SHapley Additive exPlanations; SUD, substance use disorder; TP, true positive.

### Performance vs generalized risk scoring models

The final model’s ROC AUC was higher than the parsimonious clinical model as well as LACE and Elixhauser score stratification: vs clinical baseline ΔAUC = 0.10 (95% CI, 0.07-0.13; *P* < .001); vs LACE ΔAUC = 0.19 (95% CI, 0.08-0.28; *P* < .001); vs Elixhauser ΔAUC = 0.27 (95% CI, 0.14-0.37; *P* < .001), Figure 5. Interrogating differences in risk scoring distribution vs Elixhauser and LACE risk scoring of our test cohort, both our final model and the parsimonious clinical baseline showed a conservative and right-skewed approach to emergent psychiatric presentation probability assessment, vs a qualitatively more normalized distribution for Elixhauser and LACE scoring, Figure 6A. The final model was effective in separating probabilities of those with emergent psychiatric presentations from those without emergent psychiatric presentations, ΔProbability = 0.11 (95% CI, 0.04-0.17; *P* < .001, Figure 6B).

**Figure 5.**
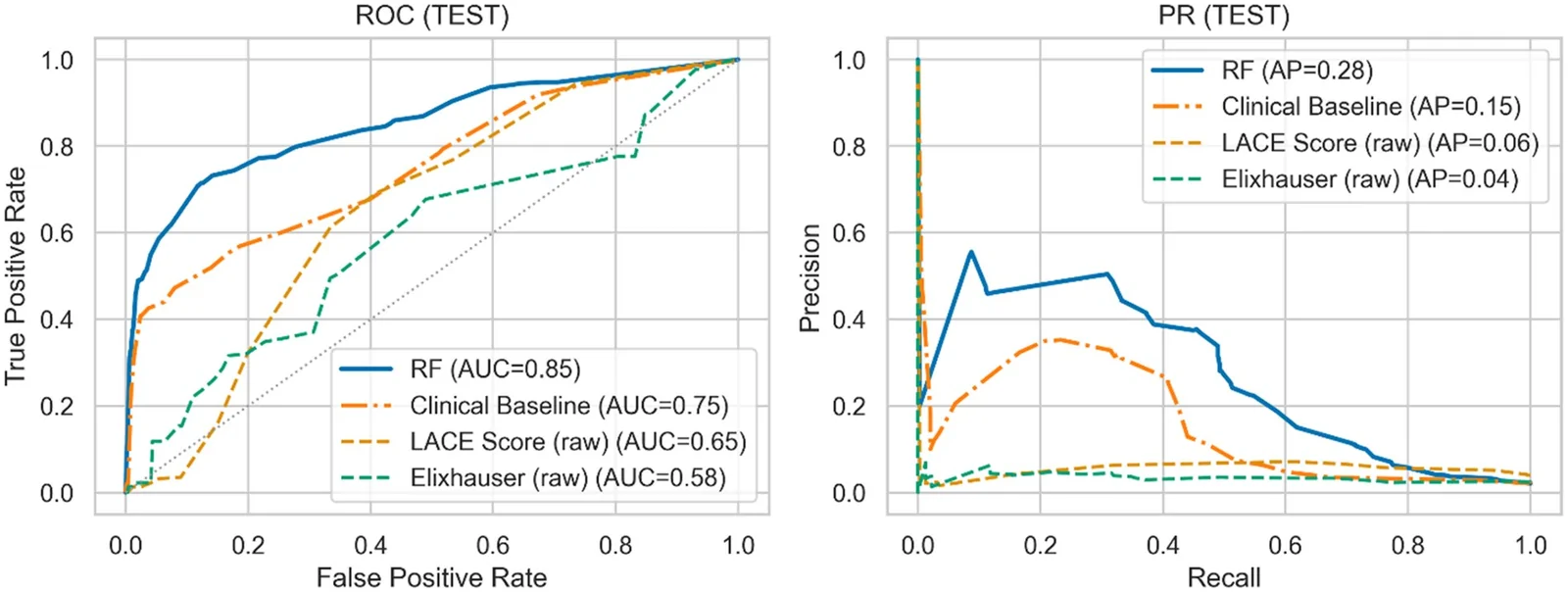
Model ROC and PR cuves. (A, left) ROC curve, optimal model vs Elixhauser and LACE scores. (B, left) PR curve, optimal model vs Elixhauser and LACE scores. Abbreviations: AP, average precision; AUC, area under the curve; PR, precision-recall; ROC, receiver-operator characteristic; RF, random forest.

**Figure 6.**
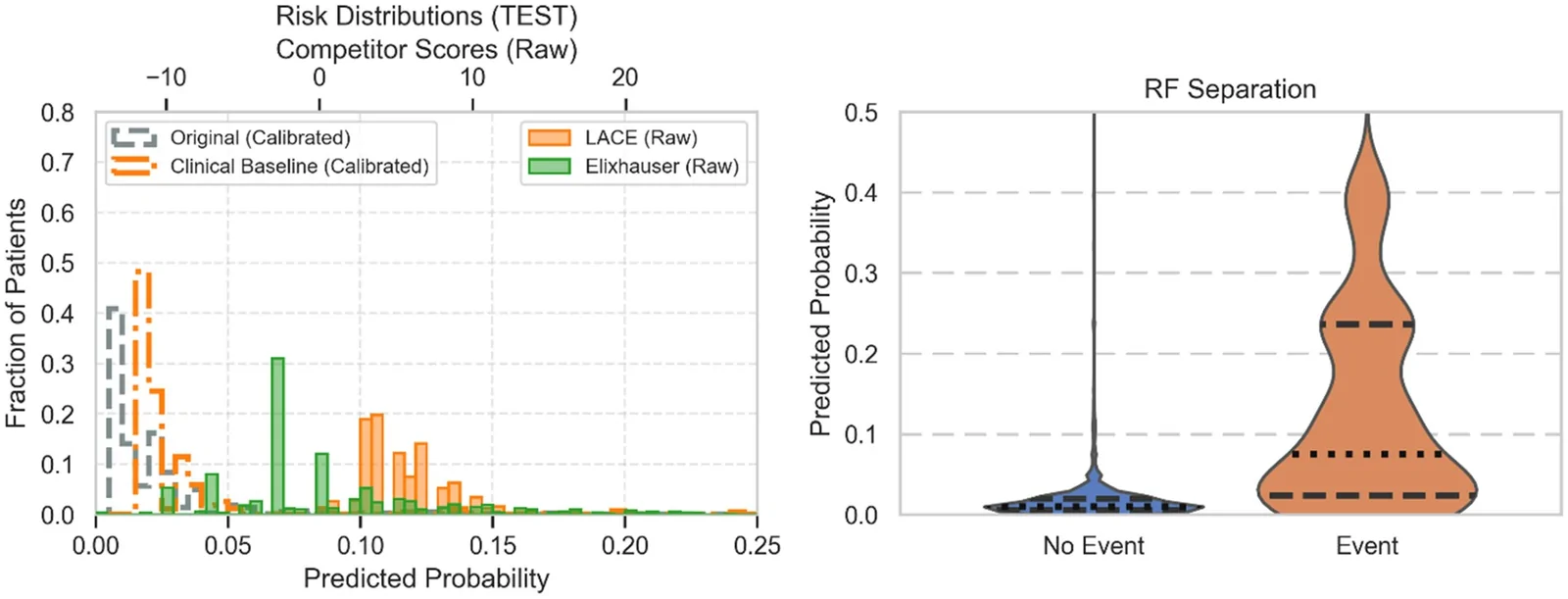
Model risk distributions vs generalized models (A) and risk distribution by event status (B). (A, left) Scoring distribution comparison, optimal model vs LACE and Elixhauser scores. Bottom *x*-axis shows predicted probability of emergent psychiatric presentation from optimal model. Top *x*-axis shows scoring of Elixhauser and LACE score comparators. (B, right) Violin plot of optimal model probability distributions. Dashed lines represent median probabilities; dotted lines represent interquartile range probabilities. Probabilities were lower (mean 0.02, SD 0.03) in patient snapshots without a 30-day emergent psychiatric presentation than those with (mean 0.13, SD 0.12) emergent psychiatric presentations. Abbreviation: RF, random forest.

## Discussion

In this retrospective cohort study of patients receiving outpatient psychiatric care, a machine learning model derived from statewide HIE data showed promising ability to predict 30-day psychiatric ED presentation in a temporally held-out test set. At a prespecified decision threshold, the model maintained 29% precision while identifying over 40% of subsequent psychiatric ED presentations. These findings suggest that structured longitudinal data aggregated across care settings could support short-term psychiatric risk stratification in outpatient practice, with potential to inform proactive outreach and care coordination for patients at elevated near-term risk of decompensation. The limited transportability of the present model should not be interpreted as limiting the broader relevance of the approach. Rather, because psychiatric emergency risk is shaped by local care patterns and data capture, the principal implication of this study is that practices which participate in statewide HIEs may be able to develop locally calibrated models that are more useful than generic risk scores for practice-specific risk stratification.

Previous work has shown that HIE use is independently associated with reduced ED length of stay and lower 30-day readmission rates, likely by bridging the information fragmentation that disproportionately affects mental health care.^20^^,^^21^ Our findings add to a growing literature on machine learning enabled prediction in mental health care, much of which has focused on suicide-related outcomes,^22–24^ treatment response,^25^ psychiatric comorbidities,^26^ and presence of substance use disorders.^27^^,^^28^ Several studies have begun to apply machine learning to psychiatric emergencies, attempting to predict ED revisits for psychiatric needs^29^ and detecting aggression among mood disorder patients.^30^ In contrast, few studies have examined near-term psychiatric ED presentation in an outpatient psychiatry population, and fewer still have shown potential for utility at a single practice level. The present results suggest that HIE-derived data may offer advantages over single-system records for this task by capturing encounters, diagnoses, and medication information across otherwise fragmented care settings. This may be particularly relevant in psychiatric care, where patients often receive services across multiple institutions and where incomplete visibility into recent utilization patterns can preclude timely intervention.

This study has several salient limitations. First, although the modeling and analytical approach described here may be adaptable to other HIE-enabled settings, the specific model developed here was derived from a single practice population and not externally validated; it may not generalize without recalibration or retraining. Second, the outcome relied on structured encounter-type fields and primary diagnosis coding, which may have misclassified some psychiatric emergencies or missed events occurring outside the HIE. Restricting the outcome to primary psychiatric or substance-related diagnoses may also have undercounted crises documented under nonpsychiatric primary codes. Third, because patients could contribute multiple snapshots, performance estimates at the snapshot level may not directly translate to patient-level alerting strategies in clinical deployment. Fourth, subgroup analyses were limited by small event counts in several strata and by missing race and ethnicity data, so fairness-related conclusions remain preliminary, and we were unable to disaggregate subtypes of psychiatric or substance use presentations. Finally, this study did not evaluate whether acting on model predictions changes patient outcomes, reduces ED use, or is cost-effective in a real-world deployment setting.

## Conclusion

In this single-practice retrospective cohort study, a locally developed machine learning model using statewide HIE data predicted 30-day psychiatric ED presentation more accurately than selected general-purpose risk scores. Although the specific model is tailored to 1 practice and should not be assumed to generalize to other settings, the findings suggest that HIE infrastructure may enable participant practices to develop locally calibrated psychiatric risk models matched to their own patient populations and care environments. Prospective studies are needed to determine whether use of such local models improves care processes or outcomes.

## Supplementary Material

ooag215_Supplementary_Data

## Data Availability

Data are available through Big Sky Care Connect with permission. J.L.H. takes responsibility for the integrity of the data and the accuracy of the analysis.

## References

[1] Patel V , Saxena S, Lund C, et al Transforming mental health systems globally: principles and policy recommendations. Lancet. 2023;402:656-666. 10.1016/S0140-6736(23)00918-2 37597892

[2] Zhu JM , Johnston KA, Hart K, et al ‘Ghost’ physicians: more than one-quarter of physicians enrolled in Medicaid delivered no care to beneficiaries in 2021. Health Aff. 2026; 45:129-137. 10.1377/hlthaff.2025.00703 PMC13035001 41628387

[3] Zhu JM , Charlesworth CJ, Polsky D, et al Phantom networks: discrepancies between reported and realized mental health care access in Oregon Medicaid. Health Aff (Millwood). 2022;41:1013-1022. 10.1377/hlthaff.2022.00052 PMC9876384 35787079

[4] Ludomirsky AB , Schpero WL, Wallace J, et al In Medicaid managed care networks, care is highly concentrated among a small percentage of physicians. Health Aff (Millwood). 2022;41:760-768. 10.1377/hlthaff.2021.01747 35500192

[5] Carlson EB , Barlow MR, Palmieri PA, et al Performance replication of the hospital mental health risk screen in ethnoracially diverse U.S. patients admitted through emergency care. PLoS One. 2024;19:e0311256. 10.1371/journal.pone.0311256 PMC11444411 39352883

[6] Austin SR , Wong YN, Uzzo RG, et al Why summary comorbidity measures such as the Charlson comorbidity index and Elixhauser score work. Med Care. 2015;53:e65-e72. 10.1097/MLR.0b013e318297429c PMC3818341 23703645

[7] Van Walraven C , Dhalla IA, Bell C, et al Derivation and validation of an index to predict early death or unplanned readmission after discharge from hospital to the community. CMAJ. 2010;182:551-557. 10.1503/cmaj.091117 PMC2845681 20194559

[8] About us—big sky care connect. Accessed February 12, 2026. https://www.mtbscc.org/about

[9] Collins GS , Moons KGM, Dhiman P, et al TRIPOD+AI statement: updated guidance for reporting clinical prediction models that use regression or machine learning methods. BMJ. 2024;385:e078378. 10.1136/bmj-2023-078378 PMC11019967 38626948

[10] Social Vulnerability Index. Place and health—geospatial research, analysis, and services program (GRASP). ATSDR. Accessed April 3, 2026. https://www.atsdr.cdc.gov/place-health/php/svi/index.html

[11] Kind AJH , Buckingham WR. Making neighborhood—disadvantage metrics accessible—the neighborhood atlas. N Engl J Med. 2018;378:2456-2458. 10.1056/NEJMP1802313 PMC6051533 29949490

[12] Hundreds of AI tools have been built to catch covid. None of them helped. MIT Technology Review. Accessed February 13, 2026. https://www.technologyreview.com/2021/07/30/1030329/machine-learning-ai-failed-covid-hospital-diagnosis-pandemic/

[13] Designing Machine Learning Systems. Chip Huyen: O'Reilly Media, 2022. Accessed February 13, 2026. https://www.oreilly.com/library/view/designing-machine-learning/9781098107956/

[14] Collins GS , Reitsma JB, Altman DG, et al Transparent reporting of a multivariable prediction model for individual prognosis or diagnosis (TRIPOD): the TRIPOD statement. BMJ. 2015;350:g7594. 10.1136/BMJ.G7594 25569120

[15] Krstajic D , Buturovic LJ, Leahy DE, et al Cross-validation pitfalls when selecting and assessing regression and classification models. J Cheminform. 2014;6:10-15. 10.1186/1758-2946-6-10 PMC3994246 24678909

[16] Wilimitis D , Walsh CG. Practical considerations and applied examples of cross-validation for model development and evaluation in health care: tutorial. JMIR AI. 2023;2:e49023. 10.2196/49023 PMC11041453 38875530

[17] Steyerberg EW. *Clinical Prediction Models*. Switzerland AG: Springer Nature, 2019, 309. 10.1007/978-3-030-16399-0

[18] DeLong ER , DeLong DM, Clarke-Pearson DL. Comparing the areas under two or more correlated receiver operating characteristic curves: a nonparametric approach. Biometrics. 1988;44:837-845. 3203132 3203132

[19] Yang YL. Holm’s sequential Bonferroni procedure. *In:* The SAGE Encyclopedia Of Educational Research, Measurement, And Evaluation. SAGE Publications, Inc, 2018, 791. 10.4135/9781506326139

[20] Ford DH , Jardim R, Landry K, et al Impact of provider health information exchange services on patient utilization of emergency room and inpatient practices in Colorado. J Clin Med. 2025;14:7783. 10.3390/jcm14217783 PMC12608124 41227177

[21] Ayer T , Ayvaci M, Karaca Z, et al The Impact of Health Information Exchanges on Emergency Department Length of Stay. Prod Oper Manag, 2018;28:740-58. 10.1111/poms.12953

[22] Walsh CG , Ribeiro JD, Franklin JC. Predicting suicide attempts in adolescents with longitudinal clinical data and machine learning. J Child Psychol Psychiatry. 2018;59:1261-1270. 10.1111/JCPP.12916 29709069

[23] De La Garza ÁG , Blanco C, Olfson M, et al Identification of suicide attempt risk factors in a national US survey using machine learning. JAMA Psychiatry. 2021;78:398-406. 10.1001/JAMAPSYCHIATRY.2020.4165 PMC7788508 33404590

[24] Choi SB , Lee W, Yoon JH, et al Ten-year prediction of suicide death using Cox regression and machine learning in a nationwide retrospective cohort study in South Korea. J Affect Disord. 2018;231:8-14. 10.1016/J.JAD.2018.01.019 29408160

[25] Chekroud AM , Bondar J, Delgadillo J, et al The promise of machine learning in predicting treatment outcomes in psychiatry. World Psychiatry. 2021;20:154-170. 10.1002/WPS.20882 PMC8129866 34002503

[26] Oliva V , De Prisco M, Pons-Cabrera MT, et al Machine learning prediction of comorbid substance use disorders among people with bipolar disorder. J Clin Med. 2022;11:3935. 10.3390/JCM11143935 PMC9315469 35887699

[27] Barenholtz E , Fitzgerald ND, Hahn WE. Machine-learning approaches to substance-abuse research: emerging trends and their implications. Curr Opin Psychiatry. 2020;33:334-342. 10.1097/YCO.0000000000000611 32304429

[28] Ahn WY , Ramesh D, Moeller FG, et al Utility of machine-learning approaches to identify behavioral markers for substance use disorders: impulsivity dimensions as predictors of current cocaine dependence. Front Psychiatry. 2016;7:34. 10.3389/fpsyt.2016.00034 PMC4785183 27014100

[29] Bians NK , Lyeo JS, Gilchrist J, et al Predicting child and adolescent mental health emergency department revisits: a machine-learning approach compared to a clinician-derived baseline. BMC Med Inform Decis Mak. 2025;26:2. 10.1186/S12911-025-03269-0 PMC12772018 41327276

[30] Lin IA , Ji X, Liu Y, et al Machine learning detecting aggression among mood disorder patients visited in psychiatric emergency departments. J Affect Disord. 2026;400:121117. 10.1016/J.JAD.2025.121117 41483885

