# Supplementary material for: A local outpatient practice-level prediction model for short-term psychiatric emergency presentation
Source: JAMIA Open. 2026 Nov 2;9(5):ooag215. doi: 10.1093/jamiaopen/ooag215 (PMC13725626; doi:10.1093/jamiaopen/ooag215)
Supplement: Supplementary file 1 [file ooag215_supplementary_data.docx]

**Methods Supplement:**

*Snapshot exclusions*

To ensure adequate historical information for feature construction, we required at least 180 days between the patient’s anchor date and the snapshot index date; snapshots not meeting this criterion were excluded. For training snapshots, feature derivation was additionally restricted to data recorded before the prespecified temporal cutoff. Moreover, to approximate a prospective deployment of our model, no snapshots from the training set occurring on or after the June 5, 2023, temporal split were included. This preserved strict temporal separation between the training and test cohorts and reduced the risk of time-correlated leakage.

*Feature engineering*

Utilization features captured prior health care use across 30-, 90-, and 365-day lookback windows, including counts of inpatient, emergency, and outpatient encounters; psychiatric-specific emergency and inpatient encounters; recency of use by setting; recent-use indicators; inpatient length of stay; utilization density; and recent escalation in service use. We also quantified care fragmentation over the prior year using continuity-of-care indices based on the distribution of encounters across providers and facilities.

Diagnostic features were based on ICD-10 codes recorded before the index date and included indicators, counts, and recency measures for any mental disorder diagnosis and for selected psychiatric subgroups, including depressive, anxiety, psychotic, and substance use disorders. Additional diagnosis-derived features captured major nonpsychiatric comorbidity clusters, including cardiovascular, pulmonary, metabolic, renal, hepatic, neurologic, infectious, musculoskeletal, and oncologic conditions.

Pharmacy features were derived from data available in BSCC including continuity of care document medication lists, First Databank data, and Medicaid claims data. Features were classified by rule-based mapping into clinically relevant classes, including antipsychotics, mood stabilizers, antidepressants, benzodiazepines, stimulants, opioids, and medications for addiction treatment. We generated class-specific indicators of recent exposure, recency of fills, and no-fill indicators over 180 days. For antipsychotics and mood stabilizers, we additionally calculated 180-day medication possession ratio and maximum gap in coverage, with derived indicators for suboptimal adherence. Additional medication features captured psychotropic polypharmacy, unique antipsychotic exposure, benzodiazepine-opioid overlap, prescriber fragmentation, and as-needed vs scheduled medication use.

Physiology and laboratory features included the most recent blood pressure and body mass index values and their recency. Laboratory features included recency of any testing, testing intensity relative to encounter volume, and the most recent available values for thyroid function, hemoglobin A1c, and lipid measures. We also derived urine drug screen positivity indicators across multiple lookback windows and recency of the last positive result when identifiable from structured result fields.

Demographic features included age, sex, race, and ethnicity. To incorporate contextual social risk, patient ZIP code prefixes were linked to Social Vulnerability Index and Area Deprivation Index reference data to derive neighborhood-level vulnerability and deprivation measures. We also identified individual-level social risk factors from ICD-10 Z-codes, including homelessness, unemployment, and problems related to primary support group. Finally, because patients could contribute multiple eligible snapshots, we included the number of prior snapshots in the preceding 180 days as a measure of observation intensity.

*Primary outcome codes*

We defined an encounter for psychiatric reasons as an encounter with a primary International Classification of Diseases, Tenth Revision (ICD-10) diagnosis codes for mental, behavioral, or neurodevelopmental disorders (F01-F99), including substance use disorders (F10-F19), and selected symptom or self-harm codes judged *a priori* to represent psychiatric emergency presentations (suicidal ideation [R45.851] and intentional self-harm codes X71-X83).

*Nested cross-validation for hyperparameter selection*

We tuned hyperparameters on and subsequently tested these classifiers’ performance on our training data in a systematic manner using a nested 5 × 5 stratified cross-validation (NCV) framework with a dependent variable of 30-day emergency psychiatric presentation, as defined above. Because patients could contribute multiple snapshots, we used patient-level grouped nested cross-validation to ensure that no patient appeared in both training and validation folds. All categorical variables were one-hot encoded. All predictors were median-imputed and standardized using a robust scaling within a preprocessing pipeline; the pipeline was refit independently inside every resampling split to prevent information leakage. Our inner five-fold cross-validation was used to tune hyperparameters and repeated a grid-search algorithm[31] and retained the best hyperparameter configuration for that fold.[32]

The inner five-fold cross-validation with grid search for hyperparameter tuning was repeated for five outer folds for each of the three classifier types tested. We conducted preliminary model testing across each of these five outer folds using hyperparameters determined from the inner five-fold cross-validation algorithm. Mean and standard deviation values across the five tuned outer folds provided an estimate of performance for each algorithm at that algorithm’s best-performing hyperparameters. We repeated this testing across our three classifiers. Of the three classifier algorithms undergoing preliminary testing, the classifier with the highest mean outer-fold precision at 33.33% recall at optimized hyperparameter settings proceeded to final model evaluation on unseen test data. We performed an identical operation on our parsimonious clinical baseline model.

*Trailing 180-day prevalence threshold adjustment:*

To adjust for recent prevalence of ED visits in the practice, we estimated the most recent 180-day training prevalence immediately prior to the temporal cutoff (π*), and aligned the calibrated decision threshold via a logit shift: threshold applied = expit(logit(threshold from cross-validation) + logit(π*) - logit(π_train)).

*Final model testing*

Once our final model was selected, we tested our hyperparameter-tuned classifier on our temporally-separated testing data, bootstrapping our data 1,000 times with replacement at the patient level to obtain confidence intervals. At the prespecified decision threshold, we recorded the number needed to evaluate and other commonly used classifier metrics of interest including number needed to evaluate, accuracy, sensitivity/recall, specificity, positive predictive value/precision, and negative predictive value. We also reported the overall receiver operator characteristic curve area under curve (ROC AUC) and precision recall curve area under curve (PR AUC) across all decision thresholds, model Brier score, and calibration slope/intercept. To test whether a few patients with many snapshots drove our results, we repeated the test-set evaluation in an additional sensitivity analysis using one random snapshot per test patient, averaged over 1,000 draws, and reported metrics estimates with confidence intervals for this analysis.

*Decision curve analysis*

Using isotonic calibrated, prior‑corrected predicted risks (the probabilities used for test decisions), we computed net benefit for each threshold as the proportion of true positives minus the proportion of false positives weighted by the harm:benefit ratio implied by the threshold (w = pt/(1-pt)); treat‑none (NB = 0) and treat‑all (NB = prevalence - (1-prevalence)·w) served as reference strategies. We plotted this net benefit as a function of the decision threshold applied, reporting net benefit at the harm:benefit ratio implied by our prespecified decision threshold.

**Supplemental Table 1:** Variables included in a 30-day psychiatric emergency prediction model.

| **Description** | **Variable type** |
| --- | --- |
| **Demographics** | |
| Age in years at index date | Continuous |
| Sex | Categorical |
| Race | Categorical |
| Ethnicity | Categorical |
| **Social Determinants of Health** | |
| Any documented homelessness | Binary indicator |
| Days since most recent documented homelessness | Continuous |
| Documented homelessness in prior 90 days | Binary indicator |
| Documented homelessness in prior 365 days | Binary indicator |
| Any documented unemployment | Binary indicator |
| Days since most recent documented unemployment | Continuous |
| Documented unemployment in prior 90 days | Binary indicator |
| Documented unemployment in prior 365 days | Binary indicator |
| Any documented primary support group problems | Binary indicator |
| Days since most recent documented primary support group problems | Continuous |
| Documented primary support group problems in prior 90 days | Binary indicator |
| Documented primary support group problems in prior 365 days | Binary indicator |
| Social Vulnerability Index, housing and transportation theme score | Continuous |
| Social Vulnerability Index, socioeconomic theme score | Continuous |
| Social Vulnerability Index, overall score | Continuous |
| Area Deprivation Index, national percentile rank | Continuous |
| Area Deprivation Index, deprivation score | Continuous |
| Social Vulnerability Index quartile | Categorical |
| **Vital Signs and Anthropometric Measures** | |
| Most recent systolic blood pressure | Continuous |
| Most recent diastolic blood pressure | Continuous |
| Days since most recent blood pressure measurement | Continuous |
| Blood pressure measured in prior 90 days | Binary indicator |
| Blood pressure measured in prior 365 days | Binary indicator |
| Most recent pulse pressure | Continuous |
| Most recent body mass index | Continuous |
| Days since most recent body mass index measurement | Continuous |
| Body mass index measured in prior 90 days | Binary indicator |
| Body mass index measured in prior 365 days | Binary indicator |
| **Healthcare Utilization** | |
| Days since most recent inpatient admission | Continuous |
| Days since most recent emergency department visit | Continuous |
| Days since most recent outpatient visit | Continuous |
| Inpatient admission in prior 90 days | Binary indicator |
| Inpatient admission in prior 365 days | Binary indicator |
| Emergency department visit in prior 90 days | Binary indicator |
| Emergency department visit in prior 365 days | Binary indicator |
| Outpatient visit in prior 90 days | Binary indicator |
| Outpatient visit in prior 365 days | Binary indicator |
| Count of inpatient admissions in prior 30 days | Continuous |
| Count of emergency department visits in prior 30 days | Continuous |
| Count of outpatient visits in prior 30 days | Continuous |
| Count of psychiatric emergency department visits in prior 30 days | Continuous |
| Count of psychiatric inpatient admissions in prior 30 days | Continuous |
| Count of inpatient admissions in prior 90 days | Continuous |
| Count of emergency department visits in prior 90 days | Continuous |
| Count of outpatient visits in prior 90 days | Continuous |
| Count of psychiatric emergency department visits in prior 90 days | Continuous |
| Count of psychiatric inpatient admissions in prior 90 days | Continuous |
| Count of inpatient admissions in prior 365 days | Continuous |
| Count of emergency department visits in prior 365 days | Continuous |
| Count of outpatient visits in prior 365 days | Continuous |
| Count of psychiatric emergency department visits in prior 365 days | Continuous |
| Count of psychiatric inpatient admissions in prior 365 days | Continuous |
| Average inpatient length of stay in prior 365 days | Continuous |
| Maximum inpatient length of stay in prior 365 days | Continuous |
| Inpatient admission rate per year (prior 365 days) | Continuous |
| Inpatient admission rate per year (all available history) | Continuous |
| Days since most recent psychiatric emergency department visit | Continuous |
| Days since most recent psychiatric inpatient admission | Continuous |
| Psychiatric emergency department visit in prior 90 days | Binary indicator |
| Psychiatric emergency department visit in prior 365 days | Binary indicator |
| Psychiatric inpatient admission in prior 90 days | Binary indicator |
| Psychiatric inpatient admission in prior 365 days | Binary indicator |
| Encounter acceleration (30-day rate relative to 365-day rate) | Continuous |
| Encounters per month (prior 365 days) | Continuous |
| Visits per month (prior 365 days) | Continuous |
| Inpatient admissions per month (prior 365 days) | Continuous |
| Emergency department visits per month (prior 365 days) | Continuous |
| Outpatient visits per month (prior 365 days) | Continuous |
| Days since first recorded encounter | Continuous |
| Ratio of inpatient utilization in prior 90 days to preceding 275 days | Continuous |
| Inpatient utilization escalation flag (prior 90 days) | Binary indicator |
| Facility continuity score (prior 365 days) | Continuous |
| **Diagnoses** | |
| Any mental health diagnosis in prior 30 days | Binary indicator |
| Any mental health diagnosis in prior 90 days | Binary indicator |
| Any mental health diagnosis in prior 365 days | Binary indicator |
| Days since most recent mental health diagnosis | Continuous |
| Mental health diagnosis recorded in prior 90 days (count) | Continuous |
| Mental health diagnosis recorded in prior 365 days (count) | Continuous |
| Any depression diagnosis in prior 30 days | Binary indicator |
| Any depression diagnosis in prior 90 days | Binary indicator |
| Any depression diagnosis in prior 365 days | Binary indicator |
| Days since most recent depression diagnosis | Continuous |
| Depression diagnosis recorded in prior 90 days (count) | Continuous |
| Depression diagnosis recorded in prior 365 days (count) | Continuous |
| Any anxiety diagnosis in prior 30 days | Binary indicator |
| Any anxiety diagnosis in prior 90 days | Binary indicator |
| Any anxiety diagnosis in prior 365 days | Binary indicator |
| Days since most recent anxiety diagnosis | Continuous |
| Anxiety diagnosis recorded in prior 90 days (count) | Continuous |
| Anxiety diagnosis recorded in prior 365 days (count) | Continuous |
| Any psychosis diagnosis in prior 30 days | Binary indicator |
| Any psychosis diagnosis in prior 90 days | Binary indicator |
| Any psychosis diagnosis in prior 365 days | Binary indicator |
| Days since most recent psychosis diagnosis | Continuous |
| Psychosis diagnosis recorded in prior 90 days (count) | Continuous |
| Psychosis diagnosis recorded in prior 365 days (count) | Continuous |
| Any substance use disorder diagnosis in prior 30 days | Binary indicator |
| Any substance use disorder diagnosis in prior 90 days | Binary indicator |
| Any substance use disorder diagnosis in prior 365 days | Binary indicator |
| Days since most recent substance use disorder diagnosis | Continuous |
| Substance use disorder diagnosis recorded in prior 90 days (count) | Continuous |
| Substance use disorder diagnosis recorded in prior 365 days (count) | Continuous |
| Any alcohol use disorder diagnosis in prior 30 days | Binary indicator |
| Any alcohol use disorder diagnosis in prior 90 days | Binary indicator |
| Any alcohol use disorder diagnosis in prior 365 days | Binary indicator |
| Days since most recent alcohol use disorder diagnosis | Continuous |
| Alcohol use disorder diagnosis recorded in prior 90 days (count) | Continuous |
| Alcohol use disorder diagnosis recorded in prior 365 days (count) | Continuous |
| Any opioid use disorder diagnosis in prior 30 days | Binary indicator |
| Any opioid use disorder diagnosis in prior 90 days | Binary indicator |
| Any opioid use disorder diagnosis in prior 365 days | Binary indicator |
| Days since most recent opioid use disorder diagnosis | Continuous |
| Opioid use disorder diagnosis recorded in prior 90 days (count) | Continuous |
| Opioid use disorder diagnosis recorded in prior 365 days (count) | Continuous |
| Any hypertension diagnosis in prior 30 days | Binary indicator |
| Any hypertension diagnosis in prior 90 days | Binary indicator |
| Any hypertension diagnosis in prior 365 days | Binary indicator |
| Days since most recent hypertension diagnosis | Continuous |
| Hypertension diagnosis recorded in prior 90 days (count) | Continuous |
| Hypertension diagnosis recorded in prior 365 days (count) | Continuous |
| Any heart failure diagnosis in prior 30 days | Binary indicator |
| Any heart failure diagnosis in prior 90 days | Binary indicator |
| Any heart failure diagnosis in prior 365 days | Binary indicator |
| Days since most recent heart failure diagnosis | Continuous |
| Heart failure diagnosis recorded in prior 90 days (count) | Continuous |
| Heart failure diagnosis recorded in prior 365 days (count) | Continuous |
| Any coronary artery disease diagnosis in prior 30 days | Binary indicator |
| Any coronary artery disease diagnosis in prior 90 days | Binary indicator |
| Any coronary artery disease diagnosis in prior 365 days | Binary indicator |
| Days since most recent coronary artery disease diagnosis | Continuous |
| Coronary artery disease diagnosis recorded in prior 90 days (count) | Continuous |
| Coronary artery disease diagnosis recorded in prior 365 days (count) | Continuous |
| Any atrial fibrillation diagnosis in prior 30 days | Binary indicator |
| Any atrial fibrillation diagnosis in prior 90 days | Binary indicator |
| Any atrial fibrillation diagnosis in prior 365 days | Binary indicator |
| Days since most recent atrial fibrillation diagnosis | Continuous |
| Atrial fibrillation diagnosis recorded in prior 90 days (count) | Continuous |
| Atrial fibrillation diagnosis recorded in prior 365 days (count) | Continuous |
| Any stroke or transient ischemic attack diagnosis in prior 30 days | Binary indicator |
| Any stroke or transient ischemic attack diagnosis in prior 90 days | Binary indicator |
| Any stroke or transient ischemic attack diagnosis in prior 365 days | Binary indicator |
| Days since most recent stroke or transient ischemic attack diagnosis | Continuous |
| Stroke or transient ischemic attack diagnosis recorded in prior 90 days (count) | Continuous |
| Stroke or transient ischemic attack diagnosis recorded in prior 365 days (count) | Continuous |
| Any chronic obstructive pulmonary disease diagnosis in prior 30 days | Binary indicator |
| Any chronic obstructive pulmonary disease diagnosis in prior 90 days | Binary indicator |
| Any chronic obstructive pulmonary disease diagnosis in prior 365 days | Binary indicator |
| Days since most recent chronic obstructive pulmonary disease diagnosis | Continuous |
| Chronic obstructive pulmonary disease diagnosis recorded in prior 90 days (count) | Continuous |
| Chronic obstructive pulmonary disease diagnosis recorded in prior 365 days (count) | Continuous |
| Any asthma diagnosis in prior 30 days | Binary indicator |
| Any asthma diagnosis in prior 90 days | Binary indicator |
| Any asthma diagnosis in prior 365 days | Binary indicator |
| Days since most recent asthma diagnosis | Continuous |
| Asthma diagnosis recorded in prior 90 days (count) | Continuous |
| Asthma diagnosis recorded in prior 365 days (count) | Continuous |
| Any diabetes mellitus diagnosis in prior 30 days | Binary indicator |
| Any diabetes mellitus diagnosis in prior 90 days | Binary indicator |
| Any diabetes mellitus diagnosis in prior 365 days | Binary indicator |
| Days since most recent diabetes mellitus diagnosis | Continuous |
| Diabetes mellitus diagnosis recorded in prior 90 days (count) | Continuous |
| Diabetes mellitus diagnosis recorded in prior 365 days (count) | Continuous |
| Any obesity diagnosis in prior 30 days | Binary indicator |
| Any obesity diagnosis in prior 90 days | Binary indicator |
| Any obesity diagnosis in prior 365 days | Binary indicator |
| Days since most recent obesity diagnosis | Continuous |
| Obesity diagnosis recorded in prior 90 days (count) | Continuous |
| Obesity diagnosis recorded in prior 365 days (count) | Continuous |
| Any hyperlipidemia diagnosis in prior 30 days | Binary indicator |
| Any hyperlipidemia diagnosis in prior 90 days | Binary indicator |
| Any hyperlipidemia diagnosis in prior 365 days | Binary indicator |
| Days since most recent hyperlipidemia diagnosis | Continuous |
| Hyperlipidemia diagnosis recorded in prior 90 days (count) | Continuous |
| Hyperlipidemia diagnosis recorded in prior 365 days (count) | Continuous |
| Any chronic kidney disease diagnosis in prior 30 days | Binary indicator |
| Any chronic kidney disease diagnosis in prior 90 days | Binary indicator |
| Any chronic kidney disease diagnosis in prior 365 days | Binary indicator |
| Days since most recent chronic kidney disease diagnosis | Continuous |
| Chronic kidney disease diagnosis recorded in prior 90 days (count) | Continuous |
| Chronic kidney disease diagnosis recorded in prior 365 days (count) | Continuous |
| Any liver disease diagnosis in prior 30 days | Binary indicator |
| Any liver disease diagnosis in prior 90 days | Binary indicator |
| Any liver disease diagnosis in prior 365 days | Binary indicator |
| Days since most recent liver disease diagnosis | Continuous |
| Liver disease diagnosis recorded in prior 90 days (count) | Continuous |
| Liver disease diagnosis recorded in prior 365 days (count) | Continuous |
| Any epilepsy diagnosis in prior 30 days | Binary indicator |
| Any epilepsy diagnosis in prior 90 days | Binary indicator |
| Any epilepsy diagnosis in prior 365 days | Binary indicator |
| Days since most recent epilepsy diagnosis | Continuous |
| Epilepsy diagnosis recorded in prior 90 days (count) | Continuous |
| Epilepsy diagnosis recorded in prior 365 days (count) | Continuous |
| Any Parkinson disease diagnosis in prior 30 days | Binary indicator |
| Any Parkinson disease diagnosis in prior 90 days | Binary indicator |
| Any Parkinson disease diagnosis in prior 365 days | Binary indicator |
| Days since most recent Parkinson disease diagnosis | Continuous |
| Parkinson disease diagnosis recorded in prior 90 days (count) | Continuous |
| Parkinson disease diagnosis recorded in prior 365 days (count) | Continuous |
| Any dementia diagnosis in prior 30 days | Binary indicator |
| Any dementia diagnosis in prior 90 days | Binary indicator |
| Any dementia diagnosis in prior 365 days | Binary indicator |
| Days since most recent dementia diagnosis | Continuous |
| Dementia diagnosis recorded in prior 90 days (count) | Continuous |
| Dementia diagnosis recorded in prior 365 days (count) | Continuous |
| Any HIV infection diagnosis in prior 30 days | Binary indicator |
| Any HIV infection diagnosis in prior 90 days | Binary indicator |
| Any HIV infection diagnosis in prior 365 days | Binary indicator |
| Days since most recent HIV infection diagnosis | Continuous |
| HIV infection diagnosis recorded in prior 90 days (count) | Continuous |
| HIV infection diagnosis recorded in prior 365 days (count) | Continuous |
| Any hepatitis C diagnosis in prior 30 days | Binary indicator |
| Any hepatitis C diagnosis in prior 90 days | Binary indicator |
| Any hepatitis C diagnosis in prior 365 days | Binary indicator |
| Days since most recent hepatitis C diagnosis | Continuous |
| Hepatitis C diagnosis recorded in prior 90 days (count) | Continuous |
| Hepatitis C diagnosis recorded in prior 365 days (count) | Continuous |
| Any sepsis diagnosis in prior 30 days | Binary indicator |
| Any sepsis diagnosis in prior 90 days | Binary indicator |
| Any sepsis diagnosis in prior 365 days | Binary indicator |
| Days since most recent sepsis diagnosis | Continuous |
| Sepsis diagnosis recorded in prior 90 days (count) | Continuous |
| Sepsis diagnosis recorded in prior 365 days (count) | Continuous |
| Any osteoarthritis diagnosis in prior 30 days | Binary indicator |
| Any osteoarthritis diagnosis in prior 90 days | Binary indicator |
| Any osteoarthritis diagnosis in prior 365 days | Binary indicator |
| Days since most recent osteoarthritis diagnosis | Continuous |
| Osteoarthritis diagnosis recorded in prior 90 days (count) | Continuous |
| Osteoarthritis diagnosis recorded in prior 365 days (count) | Continuous |
| Any rheumatoid arthritis diagnosis in prior 30 days | Binary indicator |
| Any rheumatoid arthritis diagnosis in prior 90 days | Binary indicator |
| Any rheumatoid arthritis diagnosis in prior 365 days | Binary indicator |
| Days since most recent rheumatoid arthritis diagnosis | Continuous |
| Rheumatoid arthritis diagnosis recorded in prior 90 days (count) | Continuous |
| Rheumatoid arthritis diagnosis recorded in prior 365 days (count) | Continuous |
| Any cancer diagnosis in prior 30 days | Binary indicator |
| Any cancer diagnosis in prior 90 days | Binary indicator |
| Any cancer diagnosis in prior 365 days | Binary indicator |
| Days since most recent cancer diagnosis | Continuous |
| Cancer diagnosis recorded in prior 90 days (count) | Continuous |
| Cancer diagnosis recorded in prior 365 days (count) | Continuous |
| **Medications (Pharmacy Fills)** | |
| Active antipsychotic prescription | Binary indicator |
| Antipsychotic fill in prior 90 days | Binary indicator |
| Antipsychotic fill in prior 365 days | Binary indicator |
| No antipsychotic fill in prior 180 days | Binary indicator |
| Medication possession ratio for antipsychotic (prior 180 days) | Continuous |
| Maximum gap in days in antipsychotic therapy (prior 180 days) | Continuous |
| Any gap greater than 30 days in antipsychotic therapy | Binary indicator |
| Medication possession ratio below 0.8 for antipsychotic | Binary indicator |
| Active mood stabilizer prescription | Binary indicator |
| Mood stabilizer fill in prior 90 days | Binary indicator |
| Mood stabilizer fill in prior 365 days | Binary indicator |
| No mood stabilizer fill in prior 180 days | Binary indicator |
| Medication possession ratio for mood stabilizer (prior 180 days) | Continuous |
| Maximum gap in days in mood stabilizer therapy (prior 180 days) | Continuous |
| Any gap greater than 30 days in mood stabilizer therapy | Binary indicator |
| Medication possession ratio below 0.8 for mood stabilizer | Binary indicator |
| Active antidepressant prescription | Binary indicator |
| Antidepressant fill in prior 90 days | Binary indicator |
| Antidepressant fill in prior 365 days | Binary indicator |
| No antidepressant fill in prior 180 days | Binary indicator |
| Active benzodiazepine prescription | Binary indicator |
| Benzodiazepine fill in prior 90 days | Binary indicator |
| Benzodiazepine fill in prior 365 days | Binary indicator |
| No benzodiazepine fill in prior 180 days | Binary indicator |
| Active stimulant prescription | Binary indicator |
| Stimulant fill in prior 90 days | Binary indicator |
| Stimulant fill in prior 365 days | Binary indicator |
| No stimulant fill in prior 180 days | Binary indicator |
| Active opioid prescription | Binary indicator |
| Opioid fill in prior 90 days | Binary indicator |
| Opioid fill in prior 365 days | Binary indicator |
| No opioid fill in prior 180 days | Binary indicator |
| Active medication for addiction treatment prescription | Binary indicator |
| Medication for addiction treatment fill in prior 90 days | Binary indicator |
| Medication for addiction treatment fill in prior 365 days | Binary indicator |
| No medication for addiction treatment fill in prior 180 days | Binary indicator |
| Active psychiatric polypharmacy in prior 90 days | Binary indicator |
| Count of unique antipsychotics in prior year | Count |
| Concurrent benzodiazepine and opioid use in prior 365 days | Binary indicator |
| Average medication possession ratio across drug classes | Continuous |
| Distinct medication classes per month (prior 365 days) | Continuous |
| Count of active psychiatric medications (prior 30 days) | Count |
| Count of active psychiatric medications (prior 90 days) | Count |
| Days of concurrent psychiatric medication use (prior 90 days) | Continuous |
| Count of unique prescribers (prior 365 days) | Count |
| Prescriber continuity score (prior 365 days) | Continuous |
| Any active as-needed (PRN) medication | Binary indicator |
| Ratio of as-needed (PRN) to scheduled medications | Continuous |
| **Laboratory Values** | |
| Days since most recent laboratory test of any type | Continuous |
| Any laboratory test in prior 90 days | Binary indicator |
| Any laboratory test in prior 365 days | Binary indicator |
| Laboratory tests per encounter (prior 365 days) | Continuous |
| Most recent thyroid stimulating hormone value | Continuous |
| Days since most recent thyroid stimulating hormone measurement | Continuous |
| Thyroid stimulating hormone measured in prior 90 days | Binary indicator |
| Thyroid stimulating hormone measured in prior 365 days | Binary indicator |
| Most recent hemoglobin A1c value | Continuous |
| Days since most recent hemoglobin A1c measurement | Continuous |
| Hemoglobin A1c measured in prior 90 days | Binary indicator |
| Hemoglobin A1c measured in prior 365 days | Binary indicator |
| Most recent free thyroxine (T4) value | Continuous |
| Days since most recent free thyroxine (T4) measurement | Continuous |
| Free thyroxine (T4) measured in prior 90 days | Binary indicator |
| Free thyroxine (T4) measured in prior 365 days | Binary indicator |
| Most recent free triiodothyronine (T3) value | Continuous |
| Days since most recent free triiodothyronine (T3) measurement | Continuous |
| Free triiodothyronine (T3) measured in prior 90 days | Binary indicator |
| Free triiodothyronine (T3) measured in prior 365 days | Binary indicator |
| Most recent HDL cholesterol value | Continuous |
| Days since most recent HDL cholesterol measurement | Continuous |
| Most recent triglycerides value | Continuous |
| Days since most recent triglycerides measurement | Continuous |
| Most recent LDL cholesterol value | Continuous |
| Days since most recent LDL cholesterol measurement | Continuous |
| HDL cholesterol measured in prior 90 days | Binary indicator |
| HDL cholesterol measured in prior 365 days | Binary indicator |
| Triglycerides measured in prior 90 days | Binary indicator |
| Triglycerides measured in prior 365 days | Binary indicator |
| LDL cholesterol measured in prior 90 days | Binary indicator |
| LDL cholesterol measured in prior 365 days | Binary indicator |
| Total cholesterol measured in prior 90 days | Binary indicator |
| Total cholesterol measured in prior 365 days | Binary indicator |
| Most recent total cholesterol to HDL ratio value | Continuous |
| Days since most recent total cholesterol to HDL ratio measurement | Continuous |
| Total cholesterol to HDL ratio measured in prior 90 days | Binary indicator |
| Total cholesterol to HDL ratio measured in prior 365 days | Binary indicator |
| Triglyceride to HDL ratio (most recent) | Continuous |
| **Urine Drug Screen** | |
| Any positive urine drug screen in prior 30 days | Binary indicator |
| Any positive urine drug screen in prior 90 days | Binary indicator |
| Any positive urine drug screen in prior 365 days | Binary indicator |
| Positive urine drug screen in prior 90 days (recency indicator) | Binary indicator |
| Positive urine drug screen in prior 365 days (recency indicator) | Binary indicator |
| ADI: Area Deprivation Index; BMI: body mass index; CAD: coronary artery disease; COPD: chronic obstructive pulmonary disease; ED: emergency department; HDL: high-density lipoprotein; HIV: human immunodeficiency virus; LDL: low-density lipoprotein; PRN: pro re nata (as needed); SVI: Social Vulnerability Index; T3: triiodothyronine; T4: thyroxine. Continuous features were median-imputed and standardized with a robust scaler; categorical features were one-hot encoded. All time windows are referenced to the prediction index date. | |

**Supplemental Table 2: Cross-validated model performance at optimized hyperparameters.**

| Fivefold-validated model performance comparison. | | | | |
| --- | --- | --- | --- | --- |
| **Metric** | **LR** | **RFC** | **LightGBM** | **Parsimonious Clinical Baseline** |
| **Precision at 1/3 Recall** | 0.210 | 0.220 | 0.209 | 0.165 |
| **SD** | 0.067 | 0.049 | 0.048 | 0.039 |
| **ROC AUC** | 0.770 | 0.784 | 0.778 | 0.701 |
| **SD** | 0.037 | 0.024 | 0.024 | 0.018 |
| **PR AUC** | 0.214 | 0.222 | 0.192 | 0.166 |
| **SD** | 0.079 | 0.069 | 0.050 | 0.042 |
| **PPV/Precision** | 0.087 | 0.097 | 0.089 | 0.080 |
| **SD** | 0.018 | 0.022 | 0.012 | 0.014 |
| **Sensitivity/Recall** | 0.660 | 0.644 | 0.663 | 0.524 |
| **SD** | 0.107 | 0.045 | 0.051 | 0.061 |
| **F1 Score** | 0.152 | 0.167 | 0.158 | 0.137 |
| **SD** | 0.026 | 0.033 | 0.018 | 0.019 |
| **Specificity** | 0.760 | 0.794 | 0.777 | 0.796 |
| **SD** | 0.096 | 0.060 | 0.048 | 0.061 |
| **NPV** | 0.986 | 0.986 | 0.986 | 0.981 |
| **SD** | 0.003 | 0.001 | 0.001 | 0.001 |
| **Balanced Accuracy** | 0.709 | 0.719 | 0.720 | 0.660 |
| **SD** | 0.023 | 0.020 | 0.016 | 0.012 |
| ROC: receiver operator characteristic; AUC: area under curve; SD: standard deviation from five outer folds; LR: logistic regression; RFC: random forest classifier; BRFC: balanced random forest classifier; LightGBM: light gradient boosted machine classifier. Highlight denotes top performing model for given metric. | | | | |

**Supplemental Table 3: Final Model Metrics.**

| Final model performance on temporally separated unseen test data at *a priori* decision threshold. | |
| --- | --- |
| **Metric** | **Model Performance** |
| **Precision at 1/3 Recall** | 0.294 |
| **SD** | 0.087 |
| **ROC AUC** | 0.850 |
| **SD** | 0.042 |
| **PR AUC** | 0.278 |
| **SD** | 0.111 |
| **Sensitivity/Recall** | 0.492 |
| **SD** | 0.132 |
| **F1 Score** | 0.369 |
| **SD** | 0.104 |
| **Specificity** | 0.975 |
| **SD** | 0.004 |
| **NPV** | 0.989 |
| **SD** | 0.002 |
| **Balanced Accuracy** | 0.734 |
| **SD** | 0.065 |
| ROC: receiver operator characteristic; AUC: area under curve; SD: standard deviation, 1,000x bootstrapped with replacement. | |

**Supplemental Table 4: Sensitivity analysis: one random snapshot per patient.**

| Final model performance on temporally separated unseen test data at *a priori* decision threshold; one random snapshot per patient. | |
| --- | --- |
| **Metric** | **Model Performance** |
| **Precision at 1/3 Recall** | 0.192 |
| **SD** | 0.079 |
| **ROC AUC** | 0.919 |
| **SD** | 0.059 |
| **PR AUC** | 0.133 |
| **SD** | 0.076 |
| **Sensitivity/Recall** | 0.333 |
| **SD** | 0.123 |
| **F1 Score** | 0.244 |
| **SD** | 0.088 |
| **Specificity** | 0.988 |
| **SD** | 0.003 |
| **NPV** | 0.994 |
| **SD** | 0.002 |
| **Balanced Accuracy** | 0.661 |
| **SD** | 0.061 |
| ROC: receiver operator characteristic; AUC: area under curve; SD: standard deviation, 1,000x bootstrapped with replacement. | |

**Supplemental Figure 1**: Study Sample and Exclusions.

BSCC: Big Sky Care Connect. Patients were excluded if missing a unique patient identifier, if they were aged under 18 at time of their first patient record in BSCC (“anchor date”), if their anchor date predated BSCC creation; duplicate encounters and encounters with missing dates were also excluded. From this initial sample, we split our data at the patient level into patients with anchor dates in the first 90% chronologically (before 2023-06-05) and those with anchor dates after this date.

**Supplemental Figure 2: Prevalence-adjusted subgroup analysis, model performance.**

Prevalence-adjusted analysis for age, sex, and race/ethnicity groups; event denotes a snapshot with a 30-day emergency psychiatric visit. Subgroup performance shown using prevalence‑adjusted average precision (sAP = [AP − prevalence]/[1 − prevalence]). Points show subgroup sAP with 95% patient-clustered bootstrap confidence intervals. Dashed line denotes overall sAP. Higher values indicate better performance; sAP=0 corresponds to random ranking.
